# Impact of absent crowds on technical and physical performances in the Chinese Soccer Super League

**DOI:** 10.3389/fpsyg.2022.959213

**Published:** 2022-07-28

**Authors:** Junjin Chen, Shuaishuai Zhai, Zenghui Xi, Peilun Li, Shuolin Zhang

**Affiliations:** ^1^Department of Physical Education of the Graduate School, Myongji University, Yongin, South Korea; ^2^College of Physical Education, Ludong University, Yantai, China; ^3^Department of Physical Education, Shandong University of Traditional Chinese Medicine, Yantai, China

**Keywords:** team sports, soccer, home advantage, sports performance analysis, Chinese Super League

## Abstract

**Purpose:**

Spectators have a significant impact on match performances in soccer, but to what extent crowd support contributes to the technical and physical performances remains unclear. This study aimed to (1) investigate the differences in terms of technical and physical performances with and without spectators; and (2) identify the key factors differentiating between win and loss when playing with and without the presence of an audience.

**Methods:**

Our study examined 794 performance records from 397 matches during the 2019–2020 seasons in the Chinese Soccer Super League. The least absolute shrinkage and selection operator (LASSO)-logistic regression was utilized to select significant predictors. Using an independent *t*-test and the Mann–Whitney non-parametric test explores the difference between matches with and without spectators. Key factors between win and loss were explored using univariate and multivariate logistic regression analyses.

**Results:**

Our study found that cross (*p* < 0.01, ES = −0.24), shots (*p* < 0.001, ES = −0.25), and shot accuracy (*p* < 0.05, ES = −0.18) displayed decreasing trends whereas sprint distances (*p* < 0.05, ES = 0.16) presented an increasing trend without spectators comparing with the crowd support. Moreover, the above three technical variables were the main factors differentiating between wins and losses. Similarly, team and opponent quality remained important potential factors affecting the match outcome.

**Conclusion:**

Match outcome or team performance is determined by a myriad of factors, but there are clear differences in technical and physical performances between matches with and without the presence of an audience. Similarly, our study provides a better explanation for the impact of crowd support on match performances whereby coaches can deploy players and adjust match strategies for ultimate success.

## Introduction

The consistently better performance observed by teams in various sporting contexts when playing at home is known as the “Home Advantage” (HA) (Jiménez Sánchez and Lavín, 2021). Factors that affected the documented phenomena have been paid constant attention over the past years (Pollard and Pollard, 2005; Lago-Peñas et al., 2017; Ruano and Pollard, 2022). Crowd support, territoriality, familiarity with the stadium, referee bias, and travel fatigue seem to be common consensus for home advantage in team sports (Pollard et al., 2017; Zhang et al., 2017, 2018; Ruano and Pollard, 2022). Furthermore, the majority of studies verified that crowd support and density may be the two most important factors that contribute to the home advantage in football (Pollard, 1986; Correia-Oliveira and Andrade-Souza, 2021).

The influence of home advantage on technical and physical performance has been well-documented in professional soccer. For example, home teams presented greater running demands (Aquino et al., 2018), higher total distances (Assis, 2016), and greater deceleration (Díez et al., 2021) than away teams. Similarly, playing away against strong opposition decreased team possession compared with playing at home (Lago, 2009). Recently, Travassos et al. (2022) investigated three balanced home matches from a Premier League team with 91 ball possessions in which a pass was performed into the opposition defensive area and overpassed the first defensive line. This study found that higher values of the width ratio between teams and the width of the attacking team for unsuccessful penetrative passes compared with successful penetrative passes, while a general decrease in distances and an increase in angles between attacking and defending players were observed between successful and unsuccessful penetrative passes.

There is a vast literature about HA in the Chinese Super League (CSL). For example, the existence of the HA in the CSL presented a decreasing trend from about 63.8 to 59.7% between the 2006 and 2016 seasons (Liu et al., 2019). Mao et al. (2016) suggested that HA had only trivial effects on the match outcome for upper-ranked teams when playing against upper-ranked opponents, whereas HA had positive effects on shot on target and shot accuracy for upper-ranked teams when playing with lower-ranked teams and for lower-ranked teams when facing the opposition of whatever strengths. Similarly, HA had a clear influence on ball possession and scoring first (Liu et al., 2021). Although the above-mentioned studies identified that HA had a significant impact on match performance and that spectators play a key role in winning a match in the CSL, these studies did not directly identify spectators' influence because it is difficult to design a randomized controlled trial to compare the difference between matches with and without crowd support.

The COVID-19 pandemic created a natural experimental condition to identify the direct impact of local crowds on match performance. From a global perspective, European and American top-class soccer matches played without spectators during the COVID-19 pandemic have been well-investigated. For example, shots on goal (accuracy) decreased for the home team without a crowd in the European soccer matches, and players' high-intensity running activities did not change but total distance and high-speed running distance decreased during the entire match (McCarrick et al., 2021; Rampinini et al., 2021; Wunderlich et al., 2021). Moreover, there was a reduction in HA for Serie A, but no change in HA for Serie B was reported in Brazilian Elite Soccer (Ribeiro et al., 2022). However, to the best of our knowledge, there is no investigation about the direct effects of absent crowds on match performance in the Asian football league. The 16 teams in the Chinese Soccer Super League were divided into two groups and played in a tournament-style behind closed doors during COVID-19 (Zhang et al., 2020). To minimize the risk of infection, all players, coaches, match officials, and other staff involved in the CSL had been required to stay inside a specific area during this period without any physical contact with the outside (Zhang et al., 2020). This policy counteracts the effects of crowd support, familiarity with the stadium, and travel fatigue on match performance. Thus, it is necessary to utilize this unique circumstance to determine which factors had the greatest impact on technical and physical performances in the CSL.

Based on the above rationales, this study aimed to (1) investigate the differences in terms of technical and physical performances with and without spectators; (2) identify the key factors differentiating between win and loss when playing with and without the presence of an audience. We hypothesized that there might be better performances in the technical and physical performances with crowd support compared to without spectators.

## Materials and methods

### Sample, data resource, and variables

The sample consisted of 474 performance records from 237 matches during the 2019 competitive season (with spectators) and 320 performance records from 160 matches during the 2020 competitive season (without spectators) in the CSL. These performance records were collected using a semi-automatic computerized video tracking system, Amisco Pro^®^. The validity and reliability of this system have been verified in previous studies (Di Salvo et al., 2007; Randers et al., 2010; Castellano et al., 2014).

Based on previous literature (Gong et al., 2021), seven physical performance-related parameters, 18 technical performance-related parameters, and two situational variables (team and opponent quality) were chosen in the analysis. The categories and definitions of the variables are presented in Table 1. The speed thresholds of the physical parameters were similar to those of previous reports (Bradley et al., 2009, 2016). The ethics committee approval for this study was obtained from the local university.

**Table 1:** Category and definition of the physical, technical, and situational variables.

**Physical performance-related parameters (unit): operational definition**
**Total distance (m):** distance covered in a match by all the players of a team.
**Sprint distance (m):** distance covered at a speed of over 25.1 km/h in a match by all the players of a team.
**Sprint efforts:** number of sprints (speed >25.1 km/h) in a match by all the players of a team.
**HSR distance (m):** distance covered of high-speed (19.7–25.1 km/h) running in a match by all the players of a team.
**HSR efforts:** number of high-speed (19.7–25.1 km/h) running in a match by all the players of a team.
**HIR distance (m):** high-intensity running consists of running, high-speed-running, and sprinting (running speed >14.4 km/h).
**HIR efforts:** number of high-intensity running in a match by all the players of a team.
**Technical performance-related parameters (unit): operational definition**
**Possession (%):** the duration when a team takes over the ball from the opposing team without any clear interruption as a proportion of total duration when the ball was in play
**Shots:** attempts to score a goal made with any (legal) part of the body, either on or off target.
**Shot accuracy (%):** shots on the target as a proportion of the total shots.
**Passes:** intentional played balls from one player to another.
**Pass accuracy (%):** successful passes as a proportion of the total passes.
**Forward passes:** intentional played balls from one player to another who is located in the opponent's half of the pitch.
**Forward pass accuracy (%):** successful forward passes as a proportion of the total forward passes.
**Cross:** any ball sent into the opposition team's area from a wide position.
**Cross accuracy (%):** successful crosses as a proportion of total crosses.
**Ground duel:** challenge between two players competing for ball possession above the hip height of the player who touched the ball.
**Ground duel won (%):** successful air duels as a proportion of the total air challenges.
**Air duel:** challenge between two players competing for ball possession above the hip height of the player who touched the ball.
**Air duel won (%):** successful air duels as a proportion of the total air challenges.
**Tackles:** attempt of players to get the ball from an opposition player in possession of the ball.
**Tackle won (%):** successful tackles as a proportion of the total tackles.
**Fouls:** any infringement penalized as foul play by a referee.
**Corner:** ball goes out of the play for a corner kick.
**Offside:** being caught in an offside position resulting in a free kick to the opposing team.
**Situational variables: operational definition**
**Team quality:** competitive level of the competing team according to the end-of-season ranking. A team was classified as “strong” (ranking from the 1st to 8th place) or “weak” (ranking from the 9th to 16th place).
**Opponent quality:** competitive level of the opposing team according to the end-of-season ranking. A team was classified as “strong” (ranking from the 1st to 8th place) or “weak” (ranking from the 9th to 16th place).

*The unit of the physical and technical performance-related parameters without units are in counts*.

### Statistical analysis

The logistic least absolute shrinkage and selection operator (LASSO) model is a shrinkage method that can actively select from a large and potentially multicollinear set of variables in the regression, resulting in a more relevant and interpretable set of predictors (Tibshirani, 2011). To obtain the subset of predictors, the LASSO regression analysis minimizes prediction error for a quantitative response variable by imposing a constraint on the model parameters that causes regression coefficients for some variables to shrink toward zero (Fu, 1998). Variables with a regression coefficient equal to zero after the shrinkage process are excluded from the model. In contrast, variables with nonzero regression coefficients are most strongly associated with the response variable (Tibshirani, 1997). Followed by the logistic regression procedure, our approach improves the regression performance and reduces the number of features in the final model, thus improving the interpretability of the model. Subsequently, the most significant features selected by the LASSO regression analysis were entered into the following study.

The selected variables are presented as the mean and standard deviation (SD) for data that were normally distributed and the frequencies and percentages for categorical variables. The Kolmogorov–Smirnov test was used to inspect the normality and homogeneity of variance of all the data. For two-group comparison, *p*-values were derived from an independent *t*-test to determine differences between groups with normally distributed data and a Mann–Whitney non-parametric test with other data. Cohen's *d* effect sizes and 95% confidence intervals (*CI*) were calculated (Cohen, 1988; Fritz et al., 2012). Effect sizes were interpreted as follows: ≤ 0.2 trivial, >0.2–0.6 small, >0.6–1.2 moderate, >1.2–2.0 large, >2.0–4.0 very large, and >4.0 extremely large (Hopkins et al., 2009). To examine the extent to which selected variables could be used to explain match outcome between matches with and without the presence of an audience, the association was initially evaluated using univariable logistic regression. Subsequently, multivariable logistic regression analysis with the backward stepwise method was used to explore variables that were independently associated with match outcome (Win = 1, Loss = 0). Only variables that were significant in univariable analyses were introduced into the multivariable logistic regression.

All analyses were performed using the statistical programming environment R (version 4.1.2). Specifically, the LASSO algorithm was performed using the “glmnet” package; the *t*-test and the Mann–Whitney non-parametric test were calculated by the “effect size” and “performance” package; univariable and multivariable logistic regression was performed using the “autoReg” package. All probabilities are two-tailed and *p* < 0.05 was considered statistically significant.

## Results

**Figure 1:**
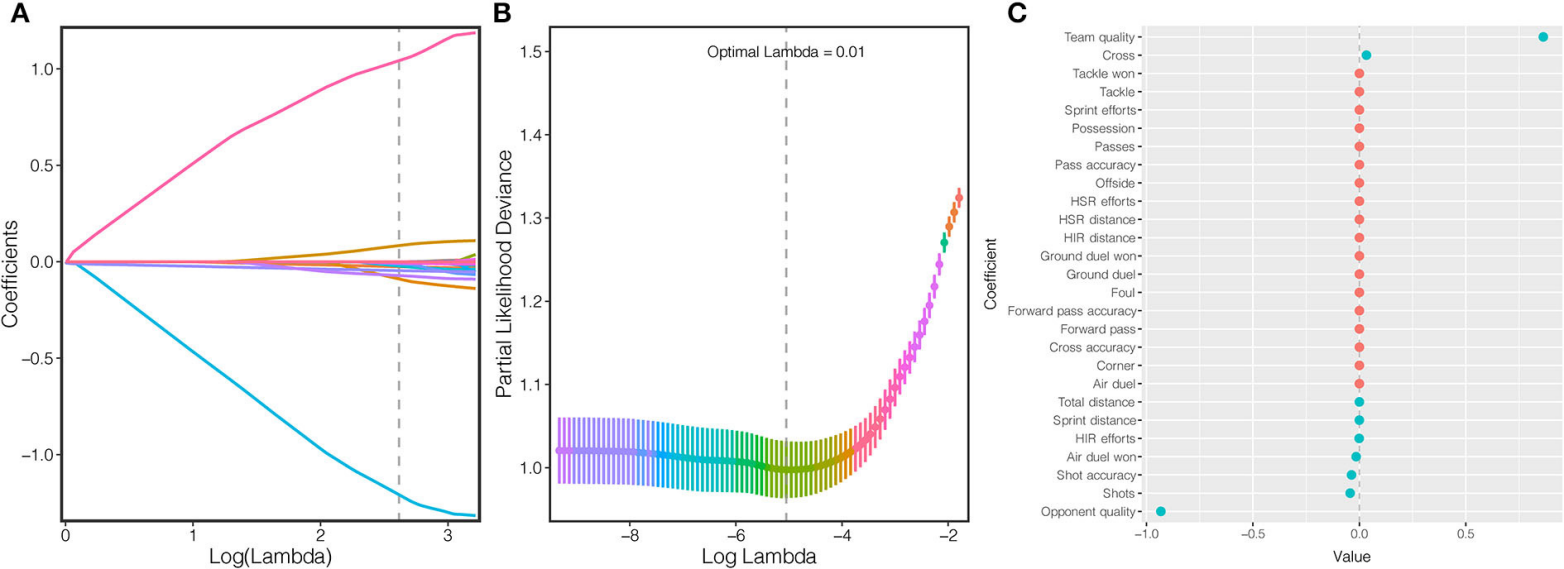
Feature selection using the least absolute shrinkage and selection operator (LASSO) binary logistic regression model. **(A)** LASSO coefficient profiles for key features, each coefficient profile plot is produced vs. log(λ) sequence. The dotted vertical line is set at the nonzero coefficients selected *via* a 10-fold cross-validation, where nine nonzero coefficients are included. **(B)** By verifying the optimal parameter (lambda) in the LASSO model, the partial likelihood deviance (binomial deviance) curve is plotted vs. log(λ). At the log (λ) of the optimal values, where features are selected, dotted vertical lines are set using the minimum criteria and the one standard error of the minimum criteria. **(C)** The specific coefficient of each variable is presented by (LASSO) binary logistic regression. The red dot means the variable is excluded while the green dot means the variable is selected in the following study.

A total of 27 features were reduced to nine potential predictors (Figure 1) with nonzero coefficients ultimately selected in the LASSO logistic regression model including four technical variables, such as cross (0.048), air dual won (−0.018), shot accuracy (−0.040), and shots (−0.056), three physical variables, such as total distance (−0.001), sprint distance (−0.001), and HIR efforts (−0.001), and two situational variables team (0.941) and opponent quality (−0.983).

**Figure 2:**
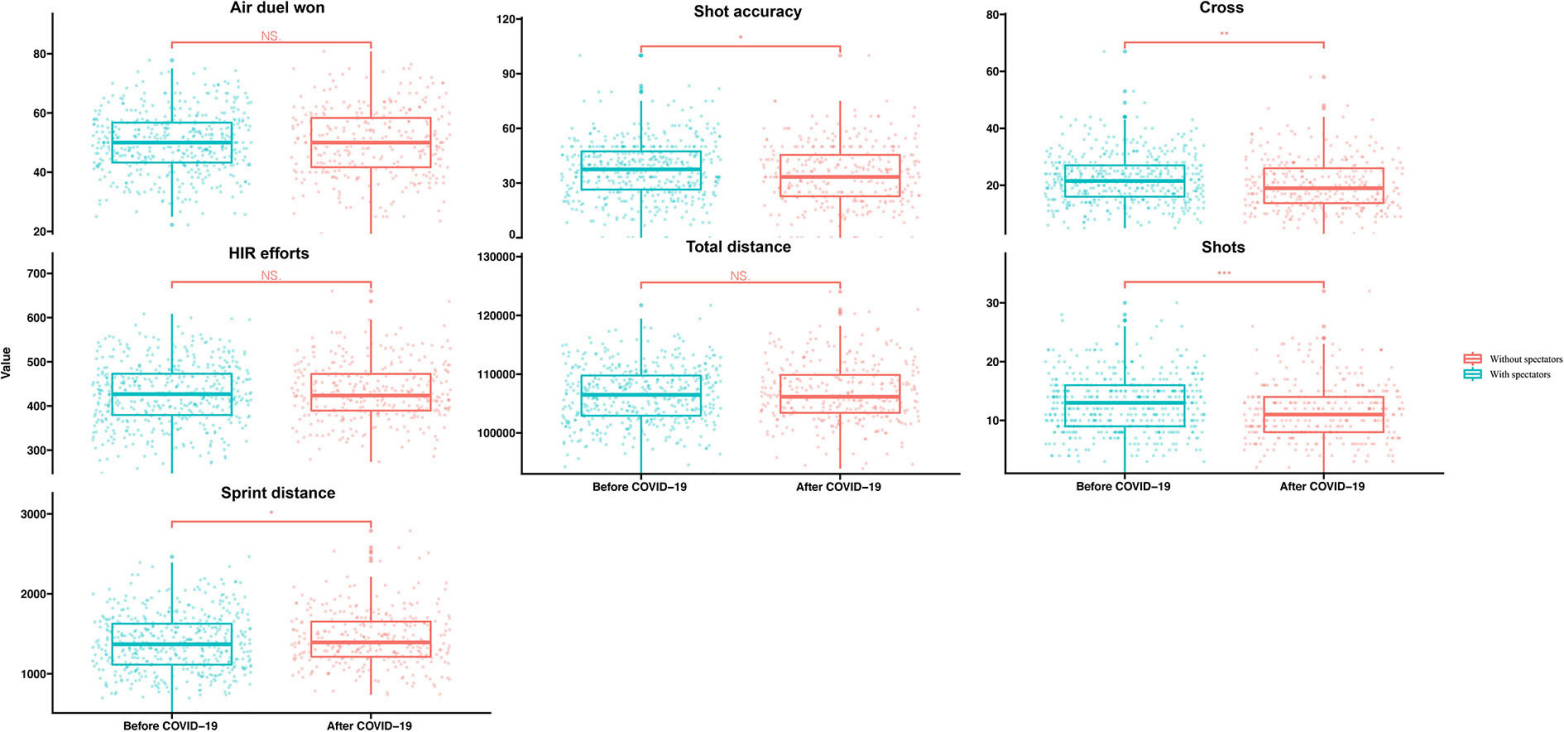
The visualization of the difference in technical and physical variables between matches with and without spectators. Note: *p < 0.05, **p < 0.01, ***p < 0.001. NS. means no statistical significance.

The match dominance indicators, such as cross (*p* < 0.01, ES = −0.24), shots (*p* < 0.001, ES = −0.25), and shot accuracy (*p* < 0.05, ES = −0.18) displayed decreasing trends without spectators comparing with the crowd support. It is worth noting that sprint distances (*p* < 0.05, ES = 0.16) presented an increasing trend without crowd support in Table 2 and Figure 2.

**Table 2:** The difference in technical and physical variables between matches with and without spectators.

**Name**	**With spectators (*N* = 474)**	**Without spectators (*N* = 320)**	***P***	**ES (95% CI)**	**Interpretation**
Cross	22.2 ± 8.5	20.1 ± 8.9	<0.01	−0.24 (−0.38, −0.10)	Small
Air duel won	50.0 ± 10.2	50.0 ± 11.6	0.995	0.01 (−0.14, 0.14)	Trivial
Shot accuracy	37.6 ± 15.8	34.8 ± 16.6	<0.05	−0.18 (−0.32, −0.03)	Trivial
Shots	12.8 ± 4.8	11.6 ± 4.9	<0.001	−0.25 (−0.39, −0.11)	Small
Total distance	106,443.5 ± 4,769.1	106,757.4 ± 5,050.3	0.375	0.06 (−0.08, 0.21)	Trivial
Sprint distance	1,386.1 ± 358.3	1,443.2 ± 337.4	<0.05	0.16 (0.02, 0.31)	Trivial
HIR efforts	427.2 ± 69.2	432.0 ± 64.9	0.329	0.07 (−0.07, 0.21)	Trivial

Univariable and multivariable analysis revealed that four technical variables were associated with winning matches in the 2019 competitive season (with spectators). These variables included both cross [odds ratio (OR), 1.08; 95% *CI*, 1.05–1.12; *p* < 0.001], air dual won (OR, 0.96; 95% CI, 0.94–0.99; *p* < 0.01), shot accuracy (OR, 0.95; 95% CI, 0.94–0.97; *p* < 0.001), and shots (OR, 0.95; 95% CI, 0.86–0.96; *p* < 0.01). In the 2020 competitive season (without spectators), three technical variables associated with winning probability, such as cross (OR, 1.08; 95% CI, 1.04–1.12; *p* < 0.001), shot accuracy (OR, 0.95; 95% CI, 0.93–0.97; *p* < 0.001), and shots (OR, 0.88; 95% CI, 0.82–0.95; *p* < 0.01). In addition, physical performance parameters had no impact on winning matches with and without spectators in Table 3.

**Table 3:** The key variables discriminated between a win and loss when playing with and without spectators.

**Indicators**	**Before COVID-19 (with spectators)**				**After COVID-19 (without spectators)**			
	**Win (*N* = 184)**	**No win (*N* = 290)**	**OR (Univariable)**	**OR (Multivariable)**	**Win (*N* = 115)**	**No win (*N* = 205)**	**OR (Univariable)**	**OR (Multivariable)**
Cross	20.7 ± 7.9	23.1 ± 8.8	1.03 (1.01–1.06, *p* = 0.004)	1.08 (1.05–1.12, *p <* 0.001)	18.3 ± 7.5	21.1 ± 9.4	1.04 (1.01–1.07, *p* = 0.008)	1.08 (1.04–1.12, *p <* 0.001)
Air duel won	52.1 ± 10.2	48.7 ± 9.9	0.97 (0.95–0.99, *p <* 0.001)	0.96 (0.94–0.99, *P* < 0.01)	52.9 ± 11.7	48.3 ± 11.3	0.97 (0.95–0.99, *p <* 0.001)	0.99 (0.96–1.01, *p* = 0.288)
Shot accuracy	44.3 ± 12.4	33.4 ± 16.3	0.95 (0.94–0.97, *p <* 0.001)	0.95 (0.94–0.97, *p <* 0.001)	42.6 ± 13.0	30.4 ± 16.8	0.95 (0.94–0.97, *p <* 0.001)	0.95 (0.93–0.97, *p <* 0.001)
Shots	13.5 ± 4.5	12.3 ± 4.9	0.95 (0.92–0.99, *p* = 0.014)	0.91 (0.86–0.96, *P* < 0.01)	12.7 ± 4.1	11.0 ± 5.2	0.93 (0.89–0.98, *p* = 0.003)	0.88 (0.82–0.95, *P* < 0.01)
Total distance	107,179.0 ± 4,681.6	105,976.9 ± 4,773.2	1.00 (1.00–1.00, *p* = 0.008)	1.00 (1.00–1.00, *p* = 0.617)	107,121.4 ± 4,874.3	106,553.1 ± 5,146.8	1.00 (1.00–1.00, *p* = 0.334)	
Sprint distance	1,465.7 ± 359.1	1,335.6 ± 349.1	1.00 (1.00–1.00, *p <* 0.001)	1.00 (1.00–1.00, *p* = 0.568)	1,471.6 ± 344.9	1,427.3 ± 333.0	1.00 (1.00–1.00, *p* = 0.261)	
HIR efforts	441.1 ± 68.8	418.4 ± 68.2	1.00 (0.99–1.00, *p <* 0.001)	1.00 (0.99–1.00, *p* = 0.209)	435.9 ± 68.5	429.8 ± 62.8	1.00 (1.00–1.00, *p* = 0.422)	
**Team quality**								
Strong	129 (70.1%)	108 (37.2%)	3.95 (2.66–5.87, *p <* 0.001)	3.43 (2.14–5.50, *p <* 0.001)	75 (65.2%)	85 (41.5%)	2.65 (1.65–4.25, *p <* 0.001)	3.05 (1.62–5.74, *p <* 0.001)
Weak	55 (29.9%)	182 (62.8%)	Reference	Reference	40 (34.8%)	120 (58.5%)	Reference	Reference
**Opponent quality**								
Strong	63 (34.2%)	174 (60%)	0.35 (0.24–0.51, *p <* 0.001)	0.29 (0.18–0.47, *p <* 0.001)	36 (31.3%)	124 (60.5%)	0.30 (0.18–0.48, *p <* 0.001)	0.18 (0.09–0.34, *p <* 0.001)
Weak	121 (65.8%)	116 (40%)	Reference	Reference	79 (68.7%)	81 (39.5%)	Reference	Reference

Strong teams have a clear advantage over weak teams to win the match in the 2019 season (OR, 3.43; 95% CI, 2.14–5.50; *p* < 0.001) and 2020 season (OR, 3.05; 95% CI, 1.62–5.74; *p* < 0.001). Moreover, compared with playing with weak teams, playing with strong teams leads to a decrease in winning probability with spectators (OR, 0.29; 95% CI, 0.18–0.47; *p* < 0.001) and without spectators (OR, 0.18; 95% CI, 0.09–0.34; *p* < 0.001) in Table 3.

## Discussion

This study aimed to (1) investigate the differences in terms of technical and physical performances with and without spectators; (2) identify the key factors differentiating between win and loss when playing with and without the presence of an audience. Our study highlighted that the match offensive indicators, such as shots, shots accuracy, and cross presented decreasing trends during match-play without spectators compared to playing with spectators. In addition, the sprint distance increased when playing without spectators, which is in contradiction with the current majority of studies from European football leagues. The determining factors of winning matches almost do not change between matches with and without crowds. Team and opponent quality remain the important factors to influence the match outcome. Our findings identify the hypothesis we made before this study.

Our study is consistent with the previous study (Almeida and Leite, 2021; Rampinini et al., 2021; Santana et al., 2021; Wunderlich et al., 2021), which found that significant decreases were observed for total shots and shots on target in five European domestic leagues (German Bundesliga, Spanish La Liga, English Premier League, Portuguese Primeira Liga, and Italian Serie A). This finding gives further evidence that these performance variables related to goal scoring were negatively affected in the European leagues as well as the Asian League (CSL). Similarly, our study is in line with the previous studies (McCarrick et al., 2021), which found that the extent of the decrease in dominance parameters, such as a cross, shots, and shots accuracy was clear during match-play when playing without spectators. Although this phenomenon can be caused for complex reasons, it indeed leads to a decreasing trend of offensive dominance during match-play without spectators. Our study speculated that social factors were deemed to be critical, due to the fans' proximity to the playing area and the more constant, loud, inspiring sounds from the crowd, where enthusiastic cheers and chants can inspire entertaining, attacking play, and encourage home players to try harder (Almeida and Leite, 2021; McCarrick et al., 2021).

Notably, our study highlighted that the overall increase in sprint distance was achieved without spectators, which is contrary to the previous study that suggested sprinting, acceleration, and deceleration distances did not change or showed a clear reduction compared to playing with spectators in the most soccer leagues (Brito de Souza et al., 2021; Rampinini et al., 2021; Santana et al., 2021; García-calvo et al., 2022). A possible explanation is that ball possessions ended without realizing the pre-prepared tactical strategies, which caused the counterattacks from the opponent. Thus, players have to cover more high-intensity running or sprint distances to recover ball possession during match-play (Raya-González et al., 2022). In general, players need several weeks of pre-season matches to achieve steady technical and tactical performances, but soccer players had difficulty with adequate preparation due to the absence of the friendly and official matches during the COVID-19 pandemic. Another possible explanation is that the Chinese football association reduced the matches during the COVID-19 lockdown and arranged all of the players into a specific area without any physical contact with the outside. This way can decrease travel fatigue, which may lead to abundant energy in physical performances applied to match-play during the limited match schedule.

Our study found that the dominance indicators, such as cross, shots, shot accuracy, and team and opponent quality are associated with match outcome whether playing with or without spectators. These results are in line with Gong et al. (2021) and Zhou et al. (2021b) that found that the dominance indicators were positively associated with match outcome and higher ball possession can increase the likelihood of shot frequencies occurring in the CSL. Based on the sustaining positive impact of the shot-related variables on winning matches, soccer coaches are required to set more practice to improve players' dribbling, precision, and tempo as well as the coordination of the crosser and the receiver to beat the offside trap (Ferraz et al., 2018; Coutinho et al., 2019). In addition, our study noted that air duals are not the key factors differentiating between win and loss when playing without spectators. Air duels in the CSL are often caused by a long pass (i.e., a header), which leads to the ball possession being reorganized as an offensive advantage (Gong et al., 2021). We suggest the coaches may change this traditional playing style and utilize more dynamic tactics to win the match without crowd support. Moreover, Zhou et al. (2021a) revealed that team and opponent quality were significant predictors associated with match outcome, especially for higher quality home teams experiencing an increased chance of winning in the CSL. Our study further identified this evidence that team and opponent quality were the potential factors that influenced match outcome, whether playing with or without crowd support.

Although the current study provided novel findings, some limitations should be acknowledged for consideration in future research. First, our study is based on the new league policy issued by the Chinese football association during the COVID-19 breakdown, which created a unique circumstance to perform the current empirical study. This policy eliminated the effects of crowd support, familiarity with the stadium, and travel fatigue on match performance, but technical and physical performances have been exclusively discussed in the current study only based on a unique factor—crowd support. Future research is recommended to explore the interactive effects of these potential factors on match performances. Second, the comparison of technical and physical performances between matches with and without crowd support had been verified in the European, American, and Asian domestic soccer leagues. Future studies could extend to African soccer leagues, the international club tournaments (e.g., UEFA Champions League), and national team events to explore more evidence. Third, future studies should seek empirical validation for our findings by applying multivariate analyses as well as new statistical techniques. A longitudinal design could be developed to perform the depth analysis.

## Conclusion

Match dominance indicators such as shots, shots accuracy, and cross presented decreasing trends during match-play without spectators, whereas the sprint distance increased when playing without spectators. In addition, shots, shots accuracy, cross, and team and opponent quality were the common factors discriminating between win and loss, whether playing with or without spectators. Match outcome or team performance is determined by a myriad of factors, but there are clear differences in technical and physical performances between matches with and without the presence of an audience.

## Data availability statement

The raw data supporting the conclusions of this article will be made available by the authors, without undue reservation.

## Ethics statement

The studies involving human participants were reviewed and approved by the Ludong University. Written informed consent from the patients/ participants or patients/participants legal guardian/next of kin was not required to participate in this study in accordance with the national legislation and the institutional requirements.

## Author contributions

JC, SZhai, and ZX contributed to the conception and design of the study. JC and SZhai collected and organized the data. PL and SZhan performed the statistical analysis. JC wrote the first draft of the manuscript. All authors contributed to the article and approved the submitted version.

## Conflict of interest

The authors declare that the research was conducted in the absence of any commercial or financial relationships that could be construed as a potential conflict of interest.

## Publisher's note

All claims expressed in this article are solely those of the authors and do not necessarily represent those of their affiliated organizations, or those of the publisher, the editors and the reviewers. Any product that may be evaluated in this article, or claim that may be made by its manufacturer, is not guaranteed or endorsed by the publisher.
